# Complete Genome Sequence of Multidrug-Resistant Strain Nocardia wallacei FMUON74, Isolated from a Sputum Culture

**DOI:** 10.1128/MRA.01022-20

**Published:** 2020-11-19

**Authors:** Masahiro Toyokawa, Makoto Taniguchi, Kazuma Uesaka, Keiko Nishimura

**Affiliations:** a Preparing Section for New Faculty of Medical Science, Fukushima Medical University, Fukushima, Fukushima, Japan; b Oral Microbiome Center, Taniguchi Dental Clinic, Takamatsu, Kagawa, Japan; c Graduate School of Bioagricultural Sciences, Nagoya University, Nagoya, Aichi, Japan; d Clinical Laboratory, Kagawa University Hospital, Kita-gun, Kagawa, Japan; University of Arizona

## Abstract

Nocardia wallacei is one of the members of the *N. transvalensis* complex which possess a highly unique susceptibility pattern. Here, we describe the closed complete genome sequence of the multidrug-resistant strain *N. wallacei* FMUON74, which was obtained using a hybrid approach combining Nanopore long-read sequencing and Illumina and DNBseq short-read sequencing.

## ANNOUNCEMENT

*Nocardia* species are ubiquitous environmental organisms that can cause opportunistic infections in humans (1). Pulmonary and disseminated infections are more prevalent in immunosuppressed patients, and the mortality rate is high (2). Nocardia wallacei is one of the members of the *N. transvalensis* complex which is generally resistant to all aminoglycosides, a highly unique susceptibility pattern among *Nocardia* species (3).

In this study, we provide a detailed description of the complete genomic sequence of the multidrug-resistant strain *N. wallacei* FMUON74, which was resistant to amikacin (MIC, >256 μg/ml), tobramycin (MIC, >256 μg/ml), clarithromycin (MIC, 16 μg/ml), imipenem (MIC, 32 μg/ml), and trimethoprim-sulfamethoxazole (MIC, 152/8 μg/ml). Antimicrobial susceptibility testing was performed using the broth microdilution method according to CLSI M24-A2 guidelines (4) using multiple panels with different lots. This strain was isolated from the sputum of a male patient with pulmonary nocardiosis. Nocardia wallacei strain FMUON74 was cultured aerobically at 37°C in tryptic soy broth from a single colony on a blood agar plate. High-molecular-weight genomic DNA was extracted with the phenol-chloroform extraction technique (5). The obtained genomic DNA was dissolved in 1/10 low Tris-EDTA (TE) buffer.

For long-read sequencing, a DNA library was prepared using a ligation sequencing kit (SQK-LSK-109; Oxford Nanopore Technologies, Ltd. [ONT], Oxford, UK) without DNA shearing and was sequenced with a GridION X5 system (ONT) on an R9.4.1 flow cell (FLO-MIN106). The long-read sequences, which were base called using Guppy v.3.6.0 (ONT), generated 127,404 reads (1,460 Mb) with an average length of 5,604 bp during a 24-h run time (numbers are for reads after quality trimming, with an average Phred quality value of >10.0 using NanoFilt v.2.7.1 [6]). For short-read sequencing, Illumina and DNBseq sequencing were performed to reduce bias. For Illumina sequencing, the paired-end Nextera DNA library (prepared using the Nextera DNA Flex library prep kit [Illumina]) was sequenced on a MiSeq instrument in 2 × 150-bp format, yielding 2,005,908 paired-end reads. For DNBseq sequencing, the MGIEasy FS PCR-free DNA library prep set (MGI Tech, Shenzhen, China) was used for the library preparation; 2 × 150-bp paired-end sequencing was performed using the DNBSEQ-G400RS FAST sequencing instrument (MGI Tech.) according to the manufacturer’s instructions, yielding 8,522,018 paired-end reads. Raw sequencing data were processed using the FASTQ preprocessing program fastp v.0.20.1 (7) for the purpose of trimming adapters and low-quality data, yielding 19.5 million short reads with an average length of 147.8 bp. Default parameters were used for all software unless otherwise specified.

For complete *de novo* genome assembly while preventing potential misassembly, we performed long-read assembly using Flye v.2.8 (8) and hybrid assembly using Unicycler v.0.4.8 (9) and compared the results. Harplot analysis of the two assembler sequences showed that no structural discrepancies exist between the two assembly sequences. Pilon v.1.23 (10) was used to polish the Unicycler assembly, generating a single circular sequence for the chromosome with a length of 7,832,428 bp (G+C content, 69.1%) and another circular sequence for a plasmid with a length of 60,434 bp (G+C content, 67.7%). To confirm that both circular contigs have no structural misassembly and no assembly gaps, we used the software program SV-Quest (https://github.com/kazumaxneo/SV-Quest), which maps short-read sequences to the contigs, detecting no signals for structural gaps and other inconsistencies. The genome was rotated to the first nucleotide of the 100 bp upstream of the *dnaA* gene. The genome sequence was then annotated using the annotation pipeline DFAST v.1.2.7.0 (11), provided by DDBJ, which predicted 7,241 coding sequences as well as 9 rRNA genes and 65 tRNA genes.

This study was conducted in accordance with the ethical guidelines of the Ministry of Health, Labor and Welfare, Japan.

### Data availability.

The closed complete chromosomal and plasmid sequences were deposited at DDBJ/EMBL/GenBank under accession numbers AP023396 and AP023397, respectively. The raw sequencing data were deposited in the DDBJ SRA database under accession numbers DRR240479 through DRR240481.
